# Mechanism of progestin resistance in endometrial precancer/cancer through Nrf2-AKR1C1 pathway

**DOI:** 10.18632/oncotarget.7004

**Published:** 2016-01-25

**Authors:** Yiying Wang, Yue Wang, Zhenbo Zhang, Ji-Young Park, Donghui Guo, Hong Liao, Xiaofang Yi, Yu Zheng, Donna Zhang, Setsuko K. Chambers, Wenxin Zheng

**Affiliations:** ^1^ Department of Obstetrics and Gynecology, Henan Province People's Hospital, Zhengzhou, China; ^2^ Department of Pathology, University of Arizona College of Medicine, Tucson, AZ, USA; ^3^ Department of Obstetrics and Gynecology, University of Arizona College of Medicine, Tucson, AZ, USA; ^4^ Reproductive Medicine, Department of Obstetrics and Gynecology, Shanghai First People's Hospital, Shanghai Jiaotong University, Shanghai, China; ^5^ Department of Pathology, Kyungpook National University, School of Medicine, Daegu, Korea; ^6^ Department of Obstetrics and Gynecology, Tianjin Gynecologic and Obstetrics Central Hospital, Tianjin, China; ^7^ Clinical and Translational Research Center, Shanghai First Maternity and Infant Hospital, Tongji University School of Medicine, Shanghai, China; ^8^ Department of Obstetrics and Gynecology, Fudan University, Shanghai, China; ^9^ Shanghai Jiai Genetics and IVF Institute, Hospital of Obstetrics and Gynecology, Fudan University, Shanghai, China; ^10^ Department of Pharmacology and Toxicology, College of Pharmacy, University of Arizona, Tucson, AZ, USA; ^11^ Department of Pathology, University of Texas Southwestern Medical Center, Dallas, TX, USA; ^12^ Arizona Cancer Center, University of Arizona, Tucson, AZ, USA

**Keywords:** Nrf2, AKR1C1, endometrial cancer, progestin resistance

## Abstract

Progestin resistance is a main obstacle for endometrial precancer/cancer conservative therapy. Therefore, biomarkers to predict progestin resistance and studies to gain a more detailed understanding of the mechanism are needed. The antioxidant Nrf2-AKR1C1 signal pathway exerts chemopreventive activity. However whether it plays a role in progestin resistance has not been explored. In this study, elevated levels of AKR1C1 and Nrf2 were found in progestin-resistant endometrial epithelia, but not in responsive endometrial glands. Exogenous overexpression of Nrf2/AKR1C1 resulted in progestin resistance. Inversely, silencing of Nrf2 or AKR1C1 rendered endometrial cancer cells more susceptible to progestin treatment. Moreover, medroxyprogesterone acetate withdrawal resulted in suppression of Nrf2/AKR1C1 expression accompanied by a reduction of cellular proliferative activity. In addition, brusatol and metformin overcame progestin resistance by down-regulating Nrf2/AKR1C1 expression. Our findings suggest that overexpression of Nrf2 and AKR1C1 in endometrial precancer/cancer may be part of the molecular mechanisms underlying progestin resistance. If validated in a larger cohort, overexpression of Nrf2 and AKR1C1 may prove to be useful biomarkers to predict progestin resistance. Targeting the Nrf2/AKR1C1 pathway may represent a new therapeutic strategy for treatment of endometrial hyperplasia/cancer.

## INTRODUCTION

Endometrial cancer is the sixth leading cause of cancer-related death in women worldwide, with the majority of cases arising in post-menopausal women [1]. However, one of the endometrial precancers, referred to as atypical hyperplasia or endometrial intraepithelial neoplasia, and well-differentiated cancer tend to occur in younger women [2–5]. Hysterectomy may not be an ideal management choice for those patients when they either have a desire to maintain their fertility or not suitable for surgery. When this happens, progestin treatment as a conservative management is commonly applied. However, approximately 30% of such patients fail to respond to progestin therapy [6]. Currently, there is no good way to identify or predict which group of patients may respond to the progestin treatment.

To better identify suitable candidates for the progestin treatment and to better understand the mechanisms of progestin resistance, significant research efforts including ours have been made in the last 2 decades to address the issue [7–12]. Down-regulation of progestin receptor (PR) resulting from continuous progestin administration leads to desensitization to progestin [13], which was thought to be one of the reasons of progestin resistance, while intermittent progestin withdrawal treatment significantly increases the apoptotic rate of endometrial cancer cells [14]. Other molecules including those in the EGF/EGFR [15, 16] and insulin [9] signaling pathways may also contribute to progestin resistance. In our earlier studies, significant decrease of survivin expression was seen in progestin responders, whereas, no significant level changes of survivin expression were seen in non-responders [7]. In a similar setting, we have also found that up-regulation of Fas/FasL expression may help response to progestin therapy in patients with endometrial hyperplasia, while dysregulation of Fas/FasL expression partially contributes to the progestin resistance [10]. It is interesting to note that Glyoxalase I (GloI) may represent another molecule involved in progestin resistance [11]. Metformin, a well-known medication for diabetes control with multiple function in cancer therapy, cell senescent and aging [17–19], could reverse progestin resistance by decreasing GloI expression [11]. Despite all these efforts and progresses observed in the past, however, the majority of the studies remain at the descriptive stage. The molecular mechanisms underlying progestin resistance remains to be demonstrated in order to improve patient care.

Mounting evidence from recent studies showed that the transcription factor NF-E2-related factor 2 (Nrf2) plays a critical role in cancer development, recurrence and resistance to adjuvant chemo- and/or radiation therapies [20–23]. The mechanisms of Nrf2 mediated drug resistance involve multiple genes and details of the molecular pathways of such drug resistance have been summarized elsewhere [20–27]. One of the Nrf2 target genes related to this study is aldoketo reductase family 1 member C1 (AKR1C1). It possesses an antioxidant response element (ARE) in the promoter region which is regulated by Nrf2. AKR1C1 is a key component of antioxidant response element in the Nrf2 signal transduction pathway [28]. It is known that the main biologic function of AKR1C1 is to convert progesterone to its inactive form, 20-alpha-dihydroxyprogesterone (20-alpha-OHP) [29, 30]. AKR1C1 also contributes to decrease local concentrations of progesterone in late secretory endometrium within the menstrual cycle [31]. The aberrant expression of AKR1C1 has been observed in endometrial cancers [30, 32, 33], and overexpression of AKR1C1 may result in an inhibition of cellular production of progestin receptor [32]. In our previous endometrial cancer studies, we showed that high level of Nrf2 expression is clearly responsible for chemoresistance [23, 34]. More importantly, brusatol, a specific inhibitor of Nrf2, could reverse chemoresistance in multiple cancers including endometrial cancer [35, 36].

In this study, we examined the role of Nrf2 and AKR1C1 in the process of progestin resistance with the following approaches: 1) to test the level of Nrf2 and AKR1C1 protein expression in progestin treated endometrial cancer samples; 2) to examine the progestin resistance through up- or down-regulation of Nrf2 and AKR1C1 expression in 2 endometrial cancer cell lines; and 3) to test if the progestin resistance could be reversed by addition of metformin and brusatol, and the effects of these agents on Nrf2 and AKR1C1 expression.

## RESULTS

### Endometrial changes, Nrf2 and AKR1C1 expression in post progestin treated endometrial samples

**Figure 1:**
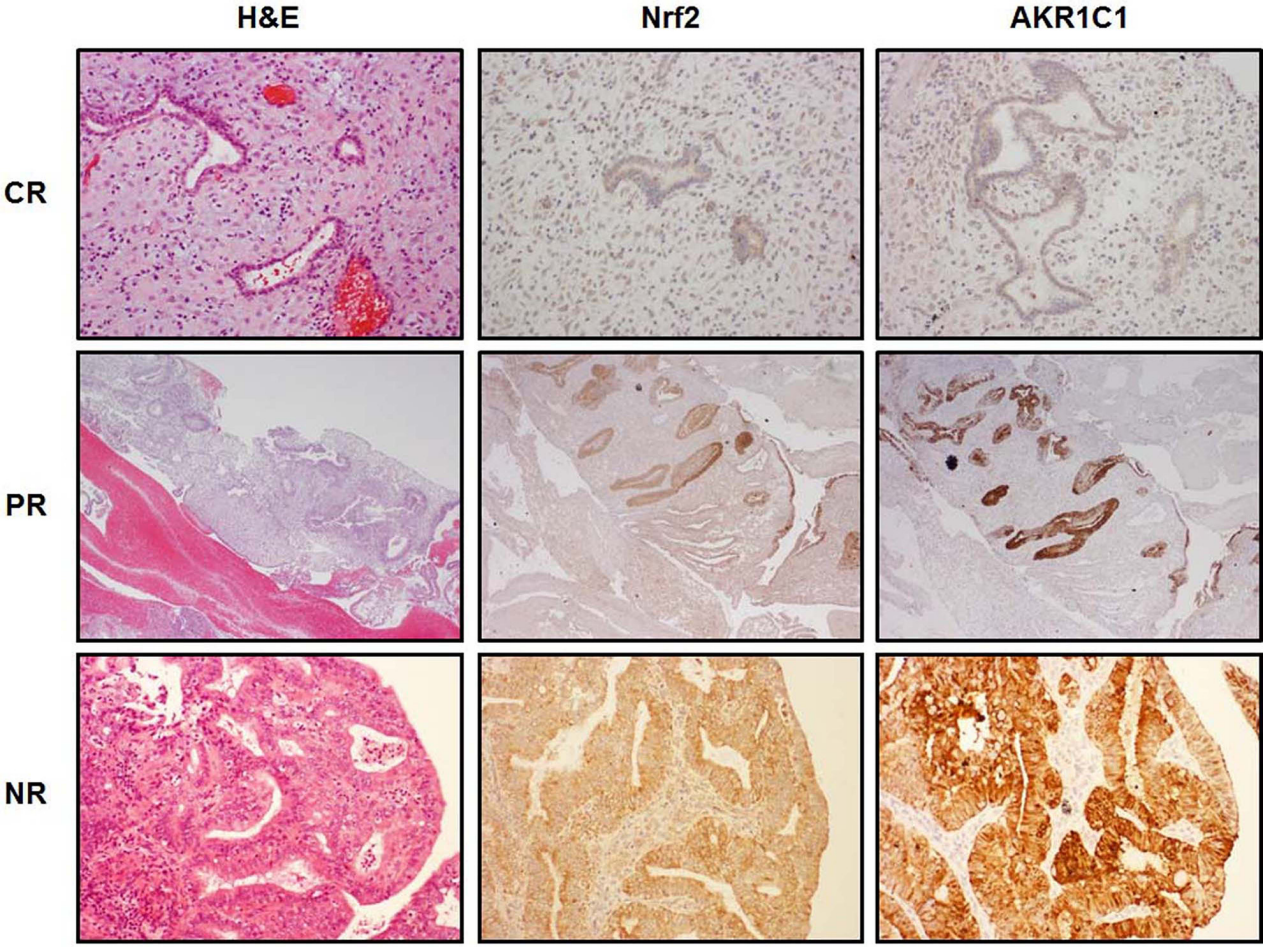
Nrf2 and AKR1C1 expression in progestin treated endometrial samples Three consecutive tissue sections were analyzed by H & E staining (left column) or by IHC for Nrf2 (middle column) and AKR1C1 (right column). Upper panel showed a complete response (CR) to progestin treatment after 6 months of MPA treatment (The original magnification: 200 ×). The middle panel represented the images of partial response (PR) to progestin treatment after 6 months of MPA treatment (The original magnification: 200 ×). Lower panel showed non-response (NR) to progestin treatment after 6 months of MPA treatment (The original magnification: 400 ×). Both Nrf2 and AKR1C1 expression were not seen in CR group, while significantly increased in PR and NR groups.

Nrf2 and AKR1C1 expression were directly correlated to the progestin resistance. In the responder group, Nrf2 and AKR1C1 were negative in all 11 post-progestin treated endometrial samples, while they were highly expressed in the samples of partial responders and non-responders (*p* < 0.001). The detailed results are summarized in Table 1 and representative pictures of Nrf2 and AKR1C1 expression are illustrated in Figure 1. Nrf2 and AKR1C1 were stained in cytoplasm and mainly found in endometrial glandular cells with only occasional stromal cell stain.

**Table 1:** Nrf2 and AKR1C1 expression in progestin treated endometrial samples

Marker scores	Responders	Partial Responders	Non-responders	*p* Values
	(*n* = 11)	(*n* = 4)	(*n* = 6)	
Nrf2	0	4.25 ± 0.63	6.67 ± 0.42	< 0.0001
AKR1C1	0	7.00 ± 0.58	9.16 ± 0.98	< 0.0001

### High levels of Nrf2 expression and progestin resistance

**Figure 2:**
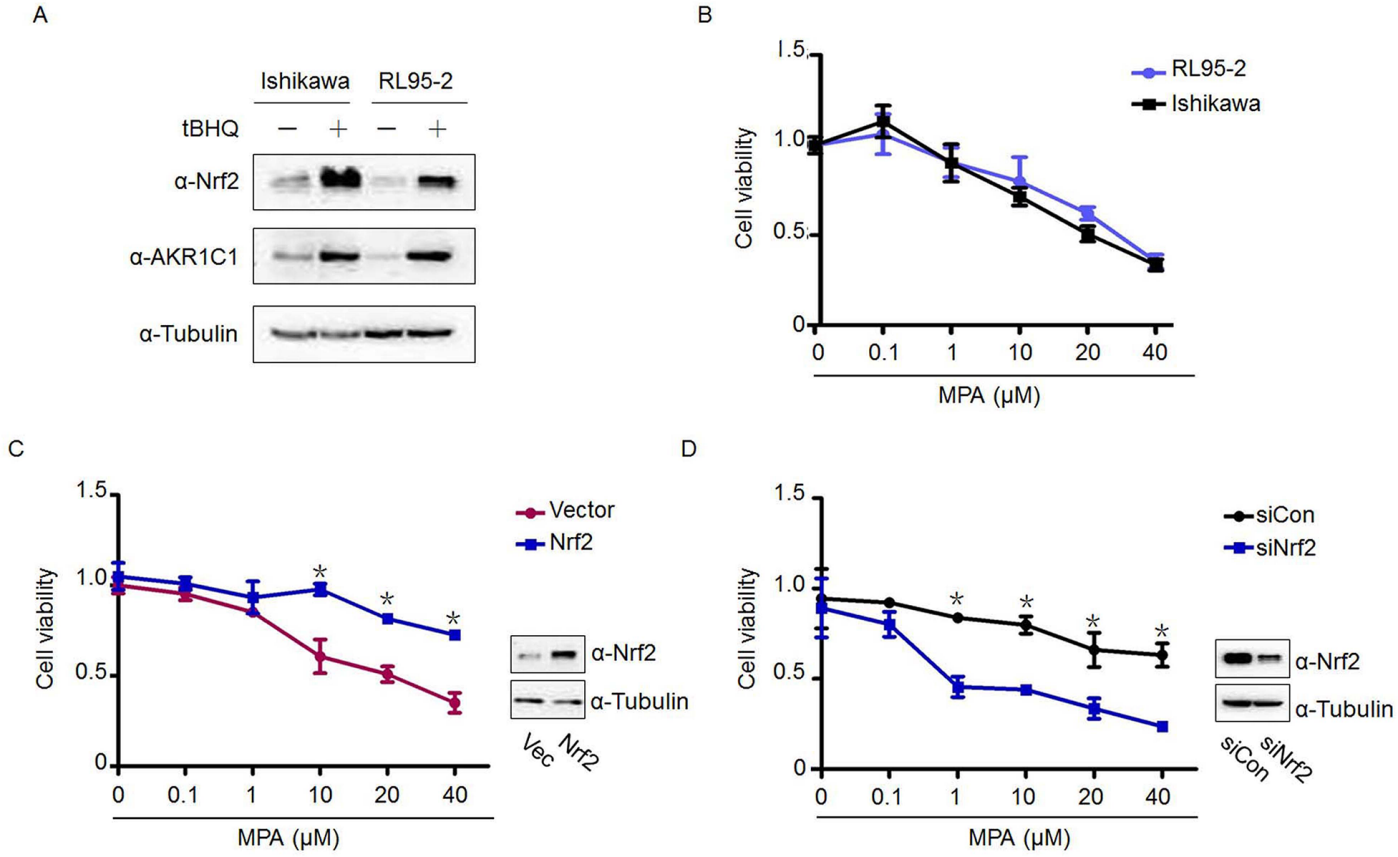
High levels of Nrf2 determine progestin resistance (**A**) The protein levels of Nrf2 and its downstream gene AKR1C1 were compared between Ishikawa and RL95–2 by Western Blot, with or without tBHQ. (**B**) MPA exerted a dose-dependent inhibitory effect on endometrial cancer proliferation (MTT assay). (**C**) Stable transfection of Nrf2 in Ishikawa cells resulted in less cell death after 48-h MPA treatment with indicated doses. Western blot was used to determine the transfection efficiency. (**D**) Silencing of Nrf2 expression by Nrf2 siRNA in Nrf2 stably transfected Ishikawa cells restored the sensitivity to 48 h MPA treatment. Cell viability was assessed by MTT assay. The silencing efficiency of Nrf2 was detected by western blot, with tubulin serving as a loading control. *p < 0.05, compared with the indicated control groups.

After observing such a striking Nrf2 and AKR1C1 expression pattern in partial and non-responding endometrial samples, we sought to investigate if these two molecules may contribute to progestin resistance in endometrial cancer. To address this issue, we first examined the level of Nrf2 expression under basal and tert-butylhydroquinone (tBHQ)-induced conditions *in vitro* by using endometrial cancer cell lines. As shown in Figure 2A, tBHQ could induce Nrf2 expression in both cell lines, with similar basal Nrf2 protein levels observed between Ishikawa and RL95–2 cells. MTT assay revealed that both cell lines responded well to progestin treatment showing a steady decline in growth activity in a dose dependent manner, with no significant difference between the cell lines found (Figure 2B). Taking into consideration that high levels of Nrf2 are reported to be related to chemotherapy resistance [24], the role of elevated Nrf2 in progestin resistance was further studied by using stably transfected cell lines. As shown in Figure 2C, overexpression of Nrf2 significantly enhanced Nrf2 protein level, which resulted in 2.4-fold decrease of cell apoptotic rate when compared with the vector-transfected control cells. The RL95–2 cells showed similar results (data not shown). Conversely, the effect of silencing of Nrf2 by siNrf2 in Nrf2 stably transfected endometrial cancer cells was evaluated. Downregulation of Nrf2 protein expression by this approach was confirmed. This resulted in a 2.1-fold sensitization of these cells to 40 μM MPA treatment (Figure 2D). Nrf2-transfected RL-95–2 cells similarly showed a 1.9-fold sensitization to progestin in the same setting (data not shown). This suggests that high levels of Nrf2 are associated with progestin resistance.

### Nrf2 associated progestin resistance through AKR1C1 mediation

**Figure 3:**
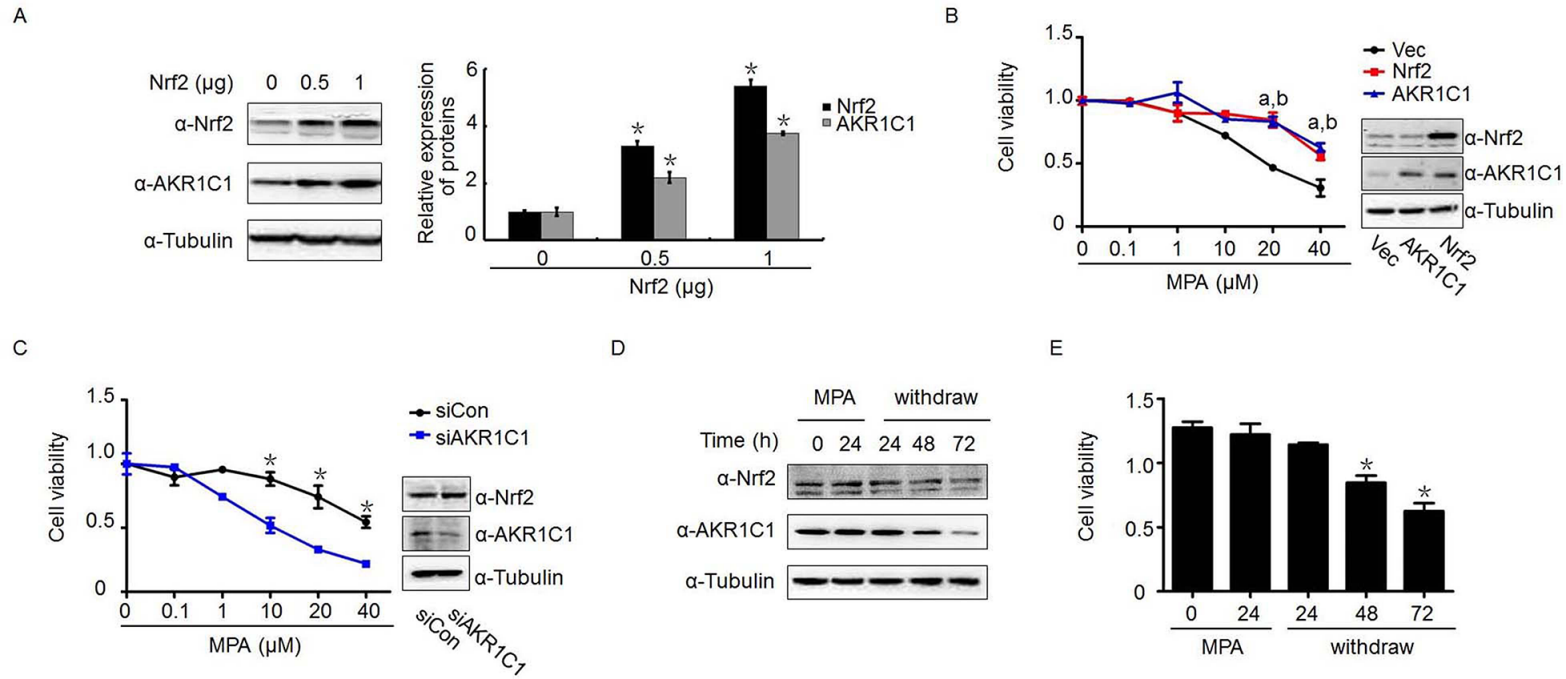
AKR1C1 mediated Nrf2-driven progestin-resistance Nrf2/AKR1C1 expression and endometrial cancer cell viability declined with progestin withdrawal. (**A**) The effect of overexpression of Nrf2 with indicated amount on Nrf2 and AKR1C1 expression. At 24 h posttransfection of plasmids, western blot was performed to determine the level of Nrf2 and AKR1C1 protein in Ishikawa cells. (**B**) Ishikawa cells were transient transfected with Nrf2 or AKR1C1 plasmid for 24 h, followed by progestin treatment with indicated dose for another 48 h. Cell viability was measured by MTT assay. a, *p* < 0.05, when Nrf2-transfected cells compared with vector-transfected group; b, *p* < 0.05, when AKRC1-transfected cells compared with vector-transfected group.(**C**) Transient transfection of siAKR1C1 in stably Nrf2-transfected Ishikawa cells for 24 h, prior to cell viability measurement, the cells were treated with indicated dose of progestin for another 48 h. Western blot was used to determine the transfection efficiency. (**D**) Effect of MPA withdrawal on Nrf2 and AKR1C1 expression in Ishikawa cells. Both proteins were significantly downregulated 72 h after the withdrawal of MPA. (**E**) MPA withdrawal for 48 and 72 h resulted in a signficant decrease in cell viability. *p < 0.05.

To explore the molecular mechanisms underlying the Nrf2-driven progestin resistance, we examined the relationship between Nrf2 and AKR1C1 since both molecules are related to drug resistance and AKR1C1 is a downstream gene of Nrf2 [29]. Consistent with the findings from IHC of this study and previous observations [29, 38], Nrf2 enhanced AKR1C1 expression in a dose-dependent manner (Figure 3A). Transient transfection of Nrf2 plasmid resulted in the elevation of both Nrf2 and AKR1C1 protein expression. Paralleling with these findings was the decreased susceptibility to progestin treatment when compared with the vector-transfected control group (Figure 3B). To clarify whether AKR1C1 is required in Nrf2-drived progestin resistance, Nrf2-transfected stable Ishikawa cells were transfected with siAKR1C1 or siCon, followed by progestin treatment. As shown in Figure 3C, despite there was no alteration of Nrf2 expression after *AKR1C1* was knocked down, transfection with siAKR1C1 profoundly increased the sensitivity to progestin treatment. It is demonstrated that the Nrf2-related progestin resistance can be corrected by reducing the level of AKR1C1 expression.

### Progestin withdrawal resulted in reduced the survival endometrial cancer cells in parallel with down regulation of Nrf2-AKR1C1

As hormone withdrawal usually causes ‘breakdown’ of the endometrium, we assessed whether removal of MPA may change the level of Nrf2 and AKR1C1 expression as well as its cell growth activity. As shown in Figure 3D, reduction of Nrf2 and AKR1C1 expression was observed starting from MPA removal at 48 h and became pronounced at 72 h. Consistent with these results, continued decline in cell proliferation with a maximal 2.1-fold decrease after 72 h of MPA removal was observed (Figure 3E).

### Downregulation of Nrf2 and AKR1C1 by brusatol and metformin to overcome progestin resistance

**Figure 4:**
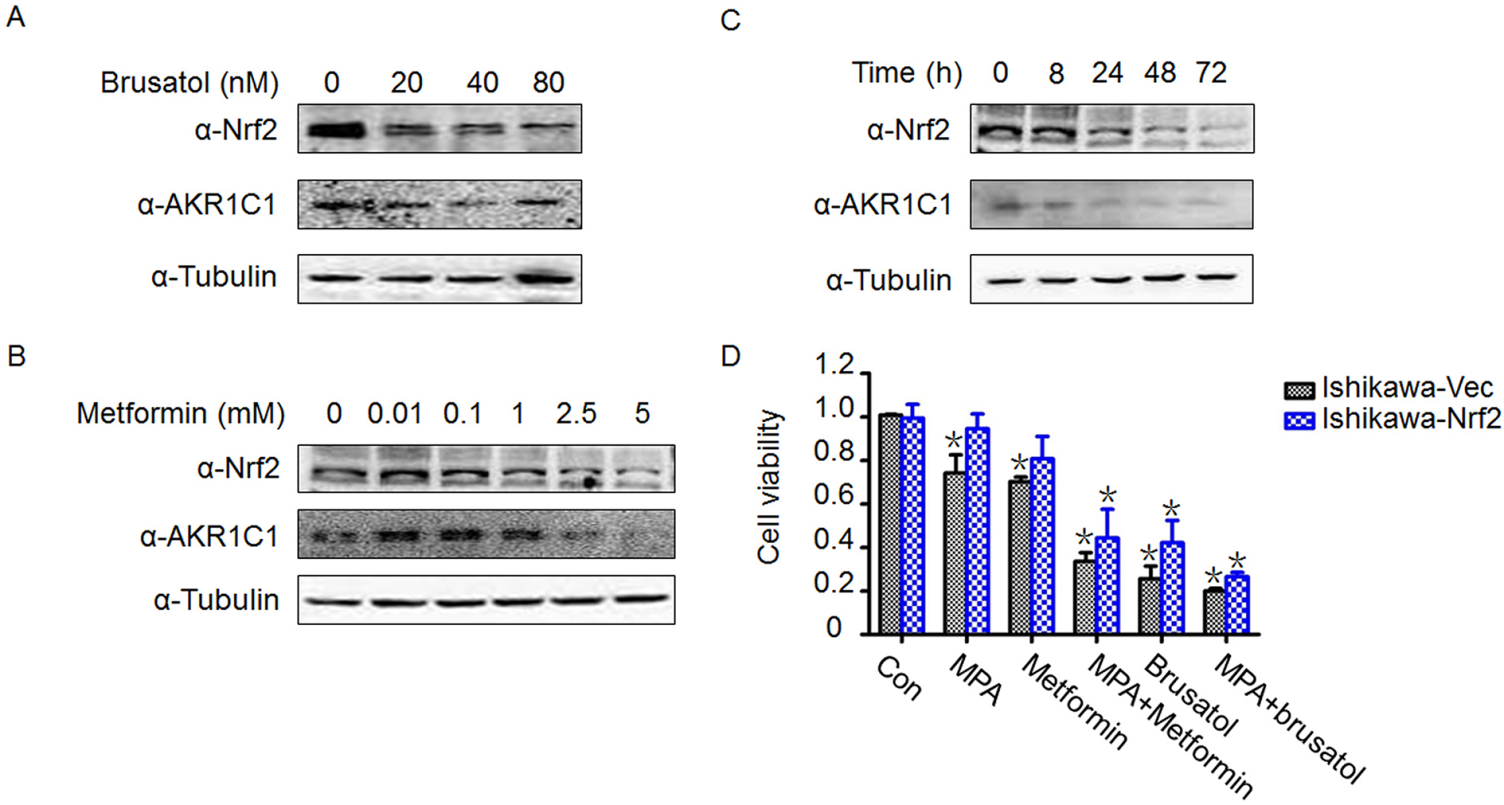
Brusatol and metformin reversed progestin resistance and downregulated Nrf2 and AKR1C1 expression (**A**) Effects of brusatol on Nrf2 and AKR1C1 expression after being treated with indicated dose of brusatol for 48 h. (**B**) Effects of metformin on Nrf2 and AKR1C1 expression after being treated with indicated dose of metformin for 48 h. (**C**) Time course of Nrf2 and AKR1C1 expression after being treated with 1 mM metformin. Ishikawa-Nrf2 cells were used in Figure A–C. (**D**) Treatment with MPA plus brusatol (20 nM) or metformin (1 mM) enhanced the sensitivity of both control Ishikawa cells and Ishikawa-Nrf2 cells to MPA administration. *p < 0.05 by student's *t*-test, when compared with each control group.

Given that Nrf2 and AKR1C1 play important roles in progestin resistance, significant therapeutic advantage may be achieved by targeting Nrf2 and its downstream genes. Our previous findings indicate that brusatol is able to enhance the efficacy of chemotherapy by inhibiting Nrf2-mediated cancer cell defense mechanism [35] and it has been shown that metformin is able to reverse progestin resistance for patients with endometrial precancers [9, 39]. Based on these understandings, we examined the role of brusatol and metformin in our experimental system to see if these molecules are potentially useful for clinical application. As shown in Figure 4A and 4B, both brusatol and metformin suppressed Nrf2 and AKR1C1 protein expression in a dose-dependent manner in Ishikawa-Nrf2 cells, similar inhibition effects also observed in Ishikawa cells (Supplementary Figure 1). In addition, metformin was able to inhibit the level of expression of the both molecules in a time-dependent fashion (Figure 4C). The inhibition time manner by brusatol was documented in our previous report [35]. The cell viability assay revealed that treatment with 10 μM MPA alone has no significant inhibition effect on progestin-resistant Ishikawa-Nrf2 cells compared with its control, whereas treatment with metformin (1 mM) plus MPA remarkably reduced the cell viability of both control and progestin-resistant cells compared with treatment with MPA or metformin individually. Compared with the effect of metformin alone, brusatol (20 nM) had a more profound inhibitory effect on both progestin sensitive and resistant cells. Strikingly, co-treatment with brusatol and MPA also led to a more pronounced decrease in cell growth compared with MPA treatment alone in both cell lines (Figure 4D). This demonstrates significant effects of both metformin and brusatol on both progestin sensitive and resistant endometrial cells. The effect was not specific to the progestin-resistant cells, as the progestin sensitive cells were also sensitized by metformin or brusatol to progestin.

## DISCUSSION

Many women in the reproductive age with endometrial precancers (atypical hyperplasia or endometrial intraepithelial neoplasia) and well-differentiated endometrial cancer have a strong desire to preserve fertility. Clinicians, however, have to follow these patients closely during the progestin conservative therapy since approximately 30% of such patients fail to respond to the progestin treatment [6]. One of the main reasons for such a high failure rate is that the molecular mechanisms of progestin resistance remain unclear. Our group has been interested in this research topic for a long time [7, 8, 11, 14]. Along the line of searching for biomarkers of prediction and revealing the molecular mechanism of progestin resistance, we recently came across the findings that Nrf2 and AKR1C1 were overexpressed only in partially responding and non-responding endometrial cancer samples after progestin treatment. In contrast to these partial or non-responders, there was no Nrf2 and AKR1C1 expression found in endometrial samples showing complete response after progestin treatment. Although the sample size of this study is relatively small, the findings are encouraging, which prompted us to search for the molecular mechanism underlying this observed progestin resistance.

In contrast to a protective role, Nrf2 has recently been shown to be related to drug resistance of human cancers [21, 24]. Interfering with Nrf2 expression by siRNA could effectively enhance sensitivity to cisplatin and arrest the cell cycle at G1 phase with a reduction of the phosphorylated form of retinoblastoma protein in lung cancer cells [21]. We previously showed that Nrf2 is overexpressed in endometrial cancer and genetic depletion of Nrf2 sensitizes endometrial cancer cells to chemotherapeutic drugs [23]. We recently provided evidence that inhibition of Nrf2 expression by brusatol could overcome chemotherapy resistance in many cancers including endometrial cancer [35]. These findings suggest that activation of Nrf2 in cancer cells provides advantages for cancer cell survival under chemotherapy pressure. However, such effect has never been explored in progestin resistance of endometrial proliferative lesions. Exploring the Nrf2 mediated molecular mechanisms was initiated when we observed striking phenomenon of Nrf2 overexpression in those non- or partially responded endometrial samples after progestin treatment. Since AKR1C1 represents a downstream gene of Nrf2 and it is readily available for a potential mechanistic study, we tested the Nrf2-AKR1C1 signal transduction pathway in this setting. One novel and intriguing finding in this study was that AKR1C1 is overexpressed in endometrial glands from partial or non-responders, but no expression in that of complete responders (Figure 1). Such observations indicate that progestin resistance may be related to the intrinsic overexpression of Nrf2-AKR1C1 signal transduction pathway, while normal or decreased Nrf2-AKR1C1 function may be indicative of good response to progestin treatment.

A similar increased level of Nrf2 and AKR1C1 expression has been reported in pancreatic cancer and such elevation may be associated with resistance to chemotherapeutic intervention [39]. It was noted that reduction of AKR1C1 by siRNA in human colon cancer enhances the sensitivity toward cisplatin, whereas overexpression of AKR1C1 is highly associated with the cisplatin resistance [26]. Similar findings were also observed in gallbladder and lung cancers [40, 41]. These results pointed out that Nrf2 and AKR1C1 contribute to the failure of drug treatment. Based on these findings, we think that the elevated AKR1C1 and Nrf2 in endometrial cancer may represent a poor prognosis due to the emergence of progestin resistance. To further investigate how Nrf2 and AKR1C1 contribute to endometrial precancer/cancer progestin resistance, we performed the mechanism related experiments *in vitro*. Transient (data not shown) or stable transfection of Nrf2 significantly attenuated the susceptibility to progestin treatment (Figure 2C). Conversely, knockdown of Nrf2 in these stable cells sensitized them to progestin treatment (Figure 2D). In terms of AKR1C1 regulation, the results paralleled those of Nrf2. Exogenous overexpression of AKR1C1 resulted in enhanced progestin resistance (Figure 3B). However, while maintaining a high level of Nrf2 in Nrf2 stably-expressed endometrial cancer cells, silencing of AKR1C1 alone abolished Nrf2-associated progestin resistance (Figure 3C). These findings indicate that Nrf2-AKR1C1 pathway may be not only responsible to the progestin resistance, but also reveals AKR1C1 being the key downstream regulator. This may be attributed to AKR1C1 special function as an enzyme to convert progesterone to its inactive form, 20-alpha-OHP [31, 43]. In addition, AKR1C1 binding with the PRB promoter and decreasing progestin-dependent PR activation [32] may also contribute to progestin resistance. This is consistent with what we observed in our previous and current studies that the level of Nrf2/AKR1C1 is persistently decreased, while PR is elevated in endometrial cancer cells after MPA withdrawal [7, 10]. It seems that the decreased level of progestin catalyzed by AKR1C1 may limit its interactions with PR and potentially contribute to the failure of progestin therapy in those non-optimal responders.

Down regulation of Nrf2/AKR1C1 is benefit to endometrial cancer cells sensitizing to progestin. As a unique inhibitor of Nrf2 expression, brusatol was examined and has been found to be able to reverse progestin resistance in the endometrial cancer cell lines with decrease of Nrf2/AKR1C1. Similar results were observed in metformin treated cells. These findings are consistent with previously demonstrated clinical observations [9] and *in vitro* experiments [43]. It is encouraging that both brusatol and metformin may be used in clinic to increase the sensitivity and efficacy of progestin treatment for those endometrial precancer or cancer patients who desire conservative therapy.

In summary, we have in this study demonstrated for the first time that Nrf2 and AKR1C1 overexpression is likely one of the main molecular mediators of progestin resistance in patients with endometrial precancers and well differentiated carcinomas. Down regulation of Nrf2 and AKR1C1 through brusatol and/or metformin application may prove to be useful to overcome progestin therapy failure. In addition, if validated, overexpression of Nrf2 and AKR1C1 may be confirmed to be useful biomarkers to predict progestin resistance.

## MATERIALS AND METHODS

### Selection of matched cases

Twenty-one post-progestin-treated endometrial hyperplasia (*n* = 15) and cancer (*n* = 6) samples were enrolled in this study. These specimens were comprised of 11 complete responders, 4 partial responders and 6 non-responders (failure to response to progestin treatment). Pathological diagnosis of endometrial hyperplasia or well-differentiated carcinoma was reviewed and confirmed by gynecologic pathologists (JYP and WZ) on the basis of WHO classification. H & E sections of endometrial samples obtained at least after 6 months of progestin administration were assessed for therapeutic responses. Patients were considered to have complete regression of hyperplasia (responders) if post progestin treated samples showed decidualized stroma with attenuated endometrial glands; If more than 50% of the entire sample contained residual hyperplasia similar or worse than the findings prior to the progestin treatment, the patient was considered to have persistent disease (non-responder); If residual hyperplastic glands comprising 50% or less, it was classified as partial response. The criteria for the responders vs non-responders were referenced based on previous publications [7, 10]. All the tissue samples were obtained from the Department of Pathology, University of Arizona and the study was approved by the Institutional Review Board.

### Tissue processing and immunohistologic (IHC) analysis

All human endometrial tissue samples were fixed in 10% buffered formalin and processed routinely for paraffin embedding. Five-micron sections for immunohistochemistry (IHC) were cut and placed on positively charged glass slides. IHC analysis of Nrf2 and AKR1C1 protein expressions was performed as previously described [7] and assessed using a semi-quantitative method [24]. Briefly, specimens were deparaffinized in xylene and rehydrated in a graded series of ethanol, subsequently, endogenous peroxidase activity was blocked by a 10-minute treatment with 3.0% hydrogen peroxide. The sections were then, after antigen retrieval, incubated overnight with rabbit anti-human Nrf2 and AKR1C1 primary antibodies at 4°C in a humid chamber, followed by a 50-minute incubation with biotinylated secondary antibody (Dako, Carpinteria, CA, USA). Omitted primary antibodies served as negative controls. Expression of Nrf2 and AKR1C1 protein was assessed using a semi-quantitative method. The stained slides were evaluated under routine microscopy for the percentage of positively stained cells (0–4) and the intensity of the staining (1–3) in the interested areas. The score of percentage is defined as follows: 0 = completely negative; 1 = less than 25% of positive cells; 2 = number of positive cells between 26–50%; 3 = number of positive cells between 51–75%, and 4 = more than 75% positive cells. Intensity is defined as follows: 1 = mild, 2 = moderate, and 3 = strong. Index of Nrf2 and AKR1C1 expression was calculated as percentage × intensity of the staining. Therefore, score 0 represents negative (−), 1–4 as weak positive (+), 5–8 as positive (++), and 9–12 as strong positive (+++). All IHC slides were reviewed independently by two investigators (Ji Young Park and WZ).

### Cell lines and cell culture

Two established progestin sensitive endometrial cancer cell lines including Ishikawa and RL95–2 were used in the experiments. The Ishikawa cell line, an estrogen-responsive cell line derived from a well-differentiated endometrioid carcinoma, was maintained in our lab. We have used it to investigate the role of Nrf2 in chemoresistance in endometrial cancer [23]. RL95–2, also derived from endometrioid carcinoma, was purchased from American Type Culture Collection (ATCC). The cells were maintained in Dulbecco's modified Eagle's medium (DMEM) F-12 1:1 medium (GIBCO) with 10% fetal bovine serum (FBS; Gibco, Gaithersburg, MD, USA), 100 U/ml penicillin, sodium pyruvate and L-glutamine in a humidified atmosphere of 5% CO_2_ at 37°C.

### Transient transfection of the cell lines and progestin administration

To investigate the roles of Nrf2 and AKR1C1 in progestin resistance, exogenous transfection of Nrf2 and AKR1C1 were performed. Briefly, after serum starvation for 24 hours, Ishikawa and RL95–2 cells were transfected with pCI-Nrf2 or pCI-AKR1C1 expression plasmids using Lipofectamine™ 3000 (Invitrogen, Carlsbad, CA, USA) according to the manufacturer's protocol. The transfection efficiency was determined by Western blot analysis. After transfection for 16 h, Nrf2- or AKR1C1-transfected cells and their corresponding controls were treated with different dose of MPA for another 48 hours. Cell proliferation was determined by the MTT assay.

### The establishment of stable cell lines, small interfering RNA transfection and progestin treatment

To identify whether silencing of AKR1C1 or Nrf2 could block Nrf2-drived progestin resistance, Ishikawa- and RL95–2 derived stable cell lines, with incorporation of Nrf2 or an empty vector, were established using retrovirus system as described previously [23, 24]. Stable Ishikawa and RL95–2 cells were continuously cultured in medium containing 1.5 μg/ml puromycin (sigma). The acute knockdown of Nrf2 or AKR1C1 in above stable cell lines was performed as previously described [23]. Briefly, cells were seeded in 0.1 ml of growth medium in 96-well plate without antibiotics, 24 hours later, transfection of Nrf2 or AKR1C1 siRNA was done according to the manufacturer's instructions with Hiperfect transfection reagent (Qiagen). After knockdown 16 h, the cells were treated with different dose of MPA for another 48 h, the cell viability was determined by MTT assay.

### Immunoblot analysis

For purpose of determining the alterations of Nrf2 and AKR1C1 after various treatments, western blots were performed as previously described [23]. The primary antibodies Nrf2, AKR1C1 and Tubulin were purchased from Santa Cruz Biotechnology. The expression patterns of Nrf2 and AKR1C1 in Ishikawa and RL95–2 were also detected by western blots with or without tBHQ treatment, which is a Nrf2 inducer, impairs the activity of the Keap1-E3 ubiquitin ligase complex through modifying the critical cysteine residues in Keap1, particularly C151, leading to stabilization of Nrf2 protein.

### Progestin, brusatol and metformin treatments

Ishikawa and RL95–2 cells were treated with 10 μM MPA for 24 h, and then withdrawed and cultured for another 24, 48 and 72 h, the cells were harvested and analyzed by Western blot to detect the changes of Nrf2 and AKR1C1 protein expression. The cells were also treated with 10 μM MPA alone, MPA plus 20 nM brusatol, or MPA plus 1 mM metformin for 48 h to examine the cell viablility and proliferative activities.

### Statistical analysis

Comparisons among multiple groups were made with one-way analysis of variance (ANOVA) followed by Dunnet *t*-test. Statistical significance between the treated and untreated groups was analyzed by Student's *t* test, and the statistical significance was set at *p* < 0.05.

## SUPPLEMENTARY MATERIALS FIGURE


