# Supplementary material for: Mechanism of progestin resistance in endometrial precancer/cancer through Nrf2-AKR1C1 pathway
Source: Oncotarget. 2016 Jan 25;7(9):10363–72. doi: 10.18632/oncotarget.7004 (PMC4891125; doi:10.18632/oncotarget.7004)
Supplement: Supplementary file 1 [file oncotarget-07-10363-s001.pdf]

Mechanism of progestin resistance in endometrial precancer/cancer through Nrf2-AKR1C1 pathway

Supplementary Materials

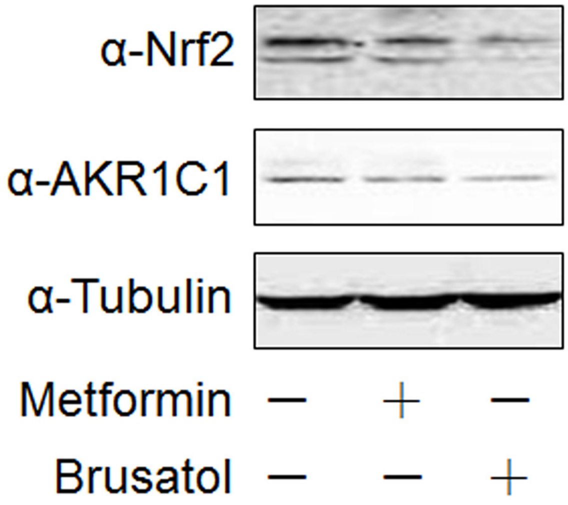

Supplementary Figure S1: The effects of metformin (1 mM) and brusatol (20 nM) on Nrf2 and AKR1C1 expressions in Ishikawa cells.
